# Three-Dimensional Reconstruction of Three-Way FRET Microscopy Improves Imaging of Multiple Protein-Protein Interactions

**DOI:** 10.1371/journal.pone.0152401

**Published:** 2016-03-29

**Authors:** Brandon L. Scott, Adam D. Hoppe

**Affiliations:** 1 Department of Chemistry and Biochemistry, South Dakota State University, Brookings, South Dakota, United States of America; 2 BioSNTR, South Dakota State University, Brookings, South Dakota, United States of America; Pennsylvania State Hershey College of Medicine, UNITED STATES

## Abstract

Fluorescence resonance energy transfer (FRET) microscopy is a powerful tool for imaging the interactions between fluorescently tagged proteins in two-dimensions. For FRET microscopy to reach its full potential, it must be able to image more than one pair of interacting molecules and image degradation from out-of-focus light must be reduced. Here we extend our previous work on the application of maximum likelihood methods to the 3-dimensional reconstruction of 3-way FRET interactions within cells. We validated the new method (3D-3Way FRET) by simulation and fluorescent protein test constructs expressed in cells. In addition, we improved the computational methods to create a 2-log reduction in computation time over our previous method (3DFSR). We applied 3D-3Way FRET to image the 3D subcellular distributions of HIV Gag assembly. Gag fused to three different FPs (CFP, YFP, and RFP), assembled into viral-like particles and created punctate FRET signals that become visible on the cell surface when 3D-3Way FRET was applied to the data. Control experiments in which YFP-Gag, RFP-Gag and free CFP were expressed, demonstrated localized FRET between YFP and RFP at sites of viral assembly that were not associated with CFP. 3D-3Way FRET provides the first approach for quantifying multiple FRET interactions while improving the 3D resolution of FRET microscopy data without introducing bias into the reconstructed estimates. This method should allow improvement of widefield, confocal and superresolution FRET microscopy data.

## Introduction

The protein-protein interactions mediate the propagation of information through biochemical signaling pathways. Fluorescence resonance energy transfer (FRET) microscopy methods are well suited for imaging the subcellular distributions of these protein-protein interactions in live cells[1]. FRET occurs when a donor’s emission spectrum and an acceptor’s excitation spectrum overlap; a requirement that comes at the cost of spectral mixing of fluorescent proteins with short Stokes shifts[2,3]. We previously developed N-Way FRET[4] to linearly unmix overlapping FRET signatures and recover the concentrations and apparent FRET efficiencies of two or more fluorophores in a single imaging plane; thereby providing a solution to spectral mixing in multifluorophore FRET microscopy. In addition to the spectral mixing, the point-spread function (PSF) of optical microscopes spatially mixes the fluorescence from neighboring volumes [5]. This situation is most severe for widefield fluorescence microscopy where the PSF is roughly three times longer in the axial (z) direction (~800 nm) than the imaging plane (xy) (~250 nm)[6]. Reconstruction/deconvolution algorithms seek to mitigate the effect of blurring by reassigning out-of-focus light to its points of origin[7]. By combining image reconstruction with spectral unmixing, the resolution of FRET microscopy can be improved greatly[8]. Here, we extend 3D FRET reconstruction to enable imaging multiple protein-protein interactions in the 3D volumes of cells.

Previously, we developed a combined spectral and spatial image reconstruction method, 3DFSR[8], on a 2-fluorophore system capable of producing high-resolution 3D maps of interacting and free molecules. That algorithm, 3DFSR, was an iterative algorithm that used alternating least squares-based line search and entropy maximization steps to generate robust estimates for the 3D concentrations and apparent FRET efficiencies of bound and free donors and acceptors. Because 3DFSR was developed using linear algebra and matrix formalism, it should be capable of reconstructing FRET data with multiple fluorophores, given the proper expansion of the spectral mixing matrix. N-Way FRET defines how this matrix should be obtained[4]. Surprisingly, our initial attempts to apply 3DFSR to 3-way FRET data from CFP, YFP, and RFP, using the spectral matrix from N-Way FRET, failed to produce accurate reconstructions. We determined the source of this limitation, developed a solution, and renamed the algorithm, 3D-3Way FRET. This new algorithm uses an alternate spectral mixing matrix for the entropy maximization step to overcome the limitations of the previous method. 3D-3Way FRET accurately recovered the 3D distributions of multiple protein-protein interactions and free molecules in living and fixed cells with improved resolution.

### Theory

The concentrations and apparent FRET efficiencies of any number of interacting fluorophores can be recovered using linear unmixing[4],

$$c=B^{-1}g$$ (1)

Where the unitized spectral mixing matrix, **B**, contains the spectral contributions of each fluorophore as well as the calibrated loss of donor fluorescence and gain of acceptor fluorescence resulting from FRET. Thus, **B** defines the relationship between the input image data vector **g** and the image vector **c** containing the fluorophore concentrations (i.e. [CFP] and [YFP]) and FRET efficiencies of the complexes (i.e. E[CFP-YFP]). Therefore, given data collected with appropriate excitation and emission combinations, an estimate of the fluorophore concentrations and apparent FRET efficiency **c** can be found by multiplying the image vector **g** by the inverse of **B**[4]. This statement forms the basis of the N-Way FRET method and is central to the development of higher order image science methods (including deconvolution) for multispectral data.

The resolution of the light microscope is finite because of the wavelike nature of light. As a result the emitted fluorescence of a single point source is not restricted to a single voxel, but rather it crosses over into multiple adjacent voxels described by the microscope PSF. Spatial mixing across voxels is described by the mathematical operation of convolution[9] between the distribution of fluorophores and the microscope’s PSF. Using linear algebra formalism, common to image science, we can express this convolution as a matrix. Thus, the spatial mixing and spectral mixing can be defined as the product of two matrices resulting in a model that captures both spatial and spectral mixing of FRET signals in 3D,

g=N(PBc+b) (2)

Where the measured data, **g**, is the product of spectral mixing, **B**, spatial mixing, **P** and the image vector **c** containing the concentrations of fluorophores and their FRET efficiencies. In practice, **g** is simply an image-vector containing the complete spectral data set required to sample all of the excitation and emission combinations required for FRET as defined in N-Way FRET. We define the contents of **c** using the same formalism as N-Way FRET; such that it contains the total FRET-corrected concentrations for each fluorophore F_i_ and the product of the FRET efficiency and the concentration of FRET complex, e.g. E_i,j_[F_i,_F_j_] such that **c =** ([F_1_], [F_2_]…E_1,2_[F_1_F_2_], E_1,3_[F_1_,F_3_]…). In addition to spatial and spectral mixing, the measured data is corrupted by Poisson noise, denoted by the function *N*, and background, **b**. Given that the noise model corrupts the data in a way that cannot be modeled by a matrix, the inversion of the PSF is an ill-posed problem[10] (i.e. effectively, **P** cannot be inverted in the presence of noise), therefore an estimate for **c** with improved resolution, cannot be obtained using linear algebra as in the 2D N-Way FRET approach. Thus iterative methods must be used.

Previous work on 3DFSR provided a solution to this problem using a single spectral matrix that was the explicit form of **B**[8]. Here an iterative approach was developed that could estimate **c** accurately and with minimal reconstruction artifacts. This approach involved combining a line search (LS) step with entropy maximization (EM) steps. Direct application of 3DFSR to the reconstruction of 3-way FRET data using an expanded three-fluorophore spectral matrix as defined in N-Way FRET[4] did not perform as well as we expected. The column sum of **B**, which normalized the data during the EM steps, contained negative column sums in 3-way FRET that were not present in the 2-way FRET version of **B**. The 3DFSR algorithm could not tolerate the negative column sums, as they imparted negative values onto the estimate of **c**, which were truncated by the non-negativity constraint. Thus, we sought to develop an improved algorithm that could overcome this limitation.

We first worked to modify the algorithm by eliminating these negative column sums. During the development of N-Way FRET, we demonstrated that the spectral contributions of each species in the data, **g**, could also be qualitatively unmixed into arbitrary units and either FRET or no FRET, **x**, as,

$$x=A^{-1}g$$ (3)

Where the spectral mixing matrix, **A**, contains only positive entries representing the fluorophore and FRET spectral signatures; however, the loss of donor fluorescence by FRET (and therefore negative values) do not explicitly appear in this form. Using matrix **A** instead of **B** eliminated the negativity issue, but in turn changed the estimates that we seek in **c** to those of **x**. Fortunately, the conversion between **x** and **c** was previously defined by N-Way FRET[4] as,

$$c={\left(\Gamma M\right)}^{-1}x$$ (4)

Where, **M** is the signed binary interaction matrix with 1s on the diagonal, and -1s where each FRET interaction creates a loss of donor fluorescence. The **Γ** matrix contains the scalar unit conversion factors on its diagonal that unitize the concentrations of each fluorophore and FRET complex contained in **c**. This conversion was used to convert between the estimates generated by the LS and EM algorithmic steps. The new 3D-3Way FRET algorithm that incorporates these changes while using our previous nomenclature[8] is presented below. We then tested 3D-3Way FRET by simulation and cellular data in live and fixed cells.

### 3D-3Way FRET algorithm

Set ***c*** = *mean*(***B***^−1^***g***).

For k = 1,2…convergence:

For each species (i) in **c**

1 $d_{1i}^{k}=P\prime \left(Pc_{i}^{k}-\;(B^{-1}g{)}_{i}\right)$ LS (preconditioned bounded line search)

Determine line search direction, *α*, with nonnegativity enforced.

2 $c_{i}^{k\left(L1\right)}=c_{i}^{k}+\;\alpha_{i}d_{1i}^{k}$
3 Enforce constraint *E*[*DA*] ≤ [*D*], *E*[*DA*] ≤ [*A*] on **c** for each FRET complex.

If ***c***_*E*[*DA*]_ > ***c***_*D*_, then ***c***_*E*[*DA*]_ = ***c***_*D*_

If ***c***_*E*[*DA*]_ > ***c***_*A*_, then ***c***_*E*[*DA*]_ = ***c***_*A*_

Convert updated concentration estimate into fluorescence abundances.

4 *x*^*k*^ = **Γ*Mc***^*k*^
5 $d_{2}^{k}=\left(\frac{A}{\sum_{n}a_{m,n}}\right)\;P\prime (\frac{g}{{\mathrm{PAx}}^{k}}-1)$ EM step + overrelaxation
6 $x^{k\;+\;1}\;=\;x^{k+1}(1+\alpha d_{2}^{k\left(L2\right)})$ (*α* determined from the likelihood functional or numerical series)

If *a* >1, refine L2 EM Step (L2 refinement–overrelaxation)

7a $d_{2}^{k\left(L2R\right)}=\left(\frac{A}{\sum_{n}a_{m,n}}\right)\;P\prime (\frac{g}{{\mathrm{PAx}}^{k}}-1)$
7b $x^{k+1}=x^{k+1}(1+\alpha d_{2}^{k\left(L2R\right)})$ (*α* = 1)

Convert the updated fluorescence abundances into concentrations and apparent FRET efficiencies.

8 ***c***^*k*+1^ = (**Γ*M***)^−1^ ***x***^*k*+1^

## Materials and Methods

### General Method for 3D-3Way FRET

Generally, the method is similar to that outlined in the N-Way and 3DFSR approaches[4,8], and can be summarized as a series of steps,

1. Capture spectral calibration images from cells expressing individual fluorophores. These data are then decomposed by parallel factor analysis to define the excitation and emission spectral fingerprints of each fluorophore in the system and the spectra of their FRET interactions. This calibration step creates the spectral fingerprint matrix **A**.
2. Capture spectral calibration images of cells expressing FRET constructs. For each FRET interaction anticipated, a linked FRET construct with FRET efficiency determined by photobleaching or fluorescence lifetime measurements is imaged. Applying the linear algebra operations defined in the N-Way FRET method will generate the unitized matrix **B** and allow estimation of the concentrations of fluorophores and apparent FRET efficiencies.
3. Determine the PSF for the microscope. The PSF can be determined by measurement by collecting 3D image stacks of 10–200 nm sized beads that are subsequently photobleach-corrected and averaged to create an average PSF or by iterative reconstruction of the PSF. Alternatively, the PSF can be computed using first principles calculations. In this study, experimental PSFs were determined and compared with theoretical PSFs. We used theoretical PSFs for reconstruction because they are perfectly smooth and symmetrical thereby simplifying the algorithm development. This step defines matrix **P** in (2).
4. Image experimental samples and reconstruct results. Here, FRET data are captured using the rule for excitation and emission bandpasses as described in N-Way FRET[4]. Data are then reconstructed using the algorithm described in the theory section.

### Constructs

Fluorescent proteins used in this study included: enhanced cyan fluorescent protein (CFP) eCFP, yellow fluorescent protein (YFP) mCitrine, red fluorescent protein (RFP) mCherry, and a FRET positive RFP-CFP-YFP construct. All the FPs used have the monomeric A206K mutations. The HIV-Gag FP fusions with CFP, YFP and RFP were described previously[4].

### Cells and Transfection

COS7 cells were obtained from the ATCC (Manassas, VA) and maintained at 37°C under 5% CO_2_ in Dulbecco’s Modified Eagle Medium (HyClone, supplemented with 10% heat-inactivated CCS, 100 U/mL penicillin and 100 μg/mL streptomycin). Cells were plated at ~3x10^5^ cells onto 25 mm round No. 1.5 coverslips (Fisherbrand) and transfected with jetPEI transfection reagent (Polyplus Transfection, Strasbourg, France) according to the manufacturers recommendations. Media was replaced 16 hours after transfection and cells were imaged 24 hours after transfection. In experiments with Gag fusion proteins, cells were fixed with 4% paraformaldehyde for 10 min prior to imaging.

### Imaging

A custom-built iMIC (FEI Munich GmbH) was used in this study and is described in detail elsewhere[4]. Briefly, the system consisted of a fast switching oligochrome module for excitation and three emCCD cameras (2 –Andor iXon 885 and 1—Andor iXonX3 885), allowing capture of fluorescence data from three fluorophores simultaneously without the need for extra excitations or filter moves to collect the excitation/emission combinations required for FRET.

### Preprocessing

All calculations were performed in Matlab (2014a, Mathworks, Natick, MA) in conjunction with the DipImage toolbox (version 2.5.1 http://www.diplib.org/, Quantitative Imaging Group, Delft University of Technology, Netherlands). All data images were preprocessed by subtracting camera bias and shade-correcting the images as previously described[5]. Briefly, images for each camera were captured while blocking all light to obtain the bias level. Residual background was subtracted from cell-free regions, if present (generally less than 5% of the cellular signal). For shade correction, images were captured of a thin solution of a fluorescent protein mixture. The measured illumination pattern across the field of view for each excitation was used to normalize bias-corrected raw data. Images were registered as described previously[11–12]. In brief, fluorescent fiducial markers (Yellow-Green PS-Speck beads, Invitrogen, Eugene, Or) immobilized on glass were simultaneously imaged on each detector to create a grid pattern to sample the field of view. A polynomial transformation vector was determined to register images, generally aligning to the yellow channel.

### Statistics

Statistical significance (p<0.05) was judged by Two-way ANOVA followed by Tukey HSD post hoc comparison of means using GraphPad Prism version 6.0e for Mac, GraphPad Software, San Diego California USA, www.graphpad.com.

*PSF measurement*. The microscope PSF was measured by collecting z-stacks (25 nm step) through 170 nm fluorescent beads (Yellow-Green PS-Speck beads) with a single bead per field-of-view. The empirical PSF was determined from the normalized average of 10 beads, and was compared with a theoretical PSF with the same size.

### Calibration

The full N-Way FRET calibration method is described in detail elsewhere[4]. Briefly, the six required excitation/emission combinations (cc, cy, cr, yy, yr, rr) were captured of cells expressing CFP, YFP, and RFP, respectively, and analyzed using the N-Way FRET algorithm to generate excitation and emission vectors by parallel factor analysis. The vectors were subsequently used to generate the spectral mixing matrix **A**. **A** contains the spectral signatures of each fluorophore (columns 1–3) and their possible FRET couplings, i.e. acceptor sensitization (columns 4–6).

$$A=\left[\begin{array}{cccccc} 1.0000 & 0.0000 & 0.0000 & 0.0020 & 0.0000 & 0.0000\\ 0.4881 & 0.3294 & 0.0000 & 1.1117 & 0.0000 & 0.0000\\ 0.0601 & 0.0228 & 0.0119 & 0.0771 & 1.1144 & 0.3302\\ 0.0000 & 0.9999 & 0.0000 & 0.0000 & 0.0000 & 0.0000\\ 0.0000 & 0.0693 & 0.0257 & 0.0000 & 0.0000 & 1.0023\\ 0.0000 & 0.0000 & 1.0000 & 0.0000 & 0.0000 & 0.0000 \end{array}\right]$$

Images of cells expressing the linked polyprotein RFP-CFP-YFP were captured to obtain the unitized unmixing matrix **B** as described in N-Way FRET. **B** contains the spectral signatures of the individual fluorophores and their unitized FRET couplings (e.g. loss in donor fluorescence and corresponding increases in acceptor fluorescence). The FRET efficiencies of RFP-CFP-YFP was determined previously by acceptor photobleaching, and used to calculate the unit conversion matrix **Γ**.

$$B=\left[\begin{array}{rrrrrr} 0.8246 & 0.0000 & 0.0000 & -0.8232 & -0.8246 & 0.0000\\ 0.4025 & 0.1653 & 0.0000 & 0.3888 & -0.4025 & -0.1653\\ 0.0495 & 0.0115 & 0.0119 & 0.0053 & 0.3586 & 0.1402\\ 0.0000 & 0.5017 & 0.0000 & 0.0000 & 0.0000 & -0.5017\\ 0.0000 & 0.0348 & 0.0257 & 0.0000 & 0.0000 & 0.4256\\ 0.0000 & 0.0000 & 1.0000 & 0.0000 & 0.0000 & 0.0000 \end{array}\right]$$

## Results and Discussion

**Fig 1:**
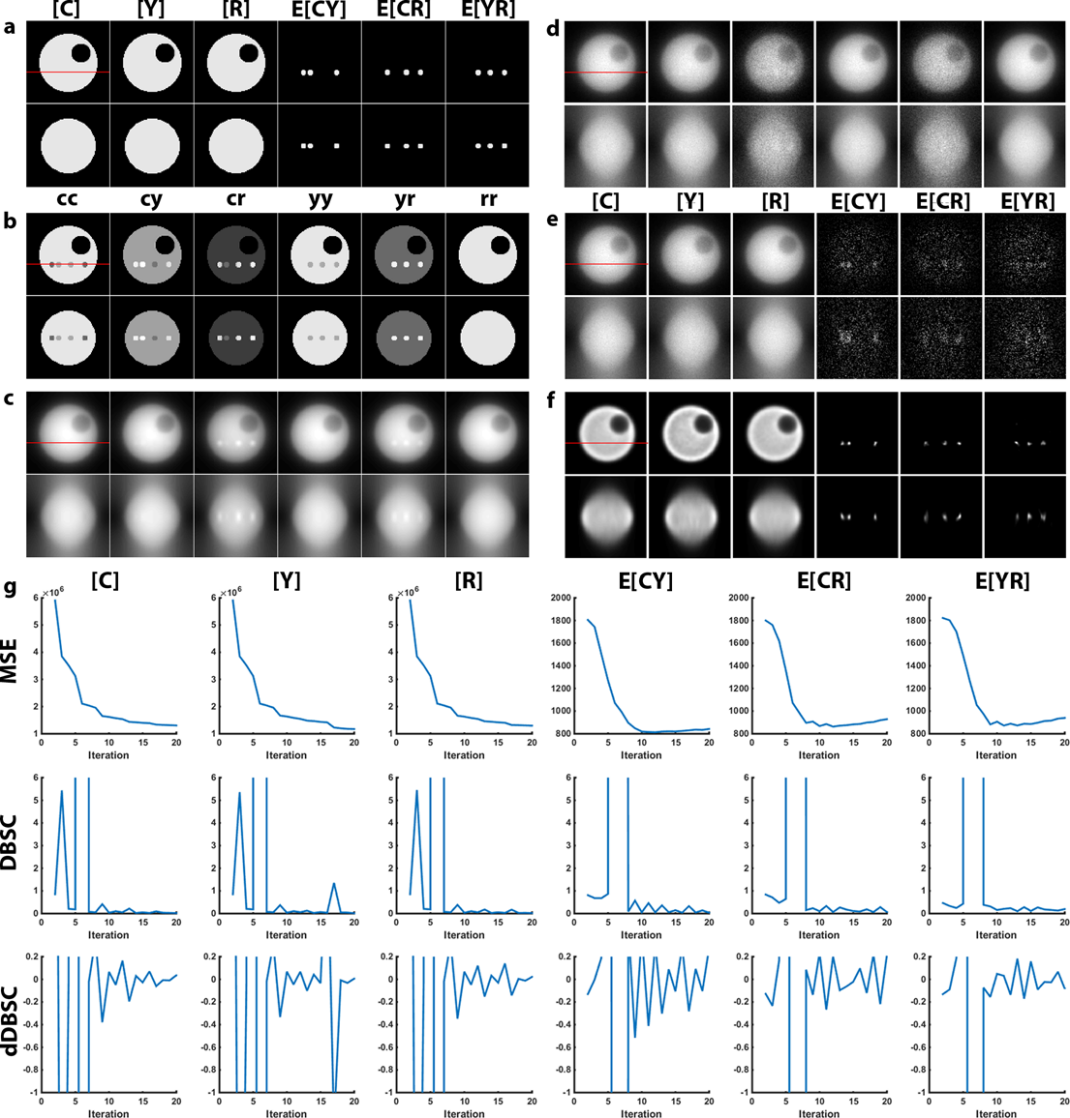
Simulation of 3D-3Way FRET data. (a) A model of a 4.5 μm diameter cell consisting of localized FRET signal within a large volume of non-FRET signals was created. The top row of images corresponds to the xy midplane of the simulated object, the red line signifies the z-plane used for the xz projection in the bottom row. (b) Spectral mixing of the fluorophore concentrations with matrix **B** (empirical) gave rise to the fluorescence detected for appropriate excitation/emission combinations (i.e. cc, cy, cr …). (c) The fluorescence distributions were blurred with a widefield PSF to account for the imperfect imaging of optical microscopes. (d) Photon noise was simulated by drawing pixel intensities from a Poisson distribution. (e) The data was directly unmixed by N-Way FRET to recover the concentration of total fluorophores and apparent FRET efficiencies, but no improvements in the optical sectioning. (f) The reconstructed estimate was generated following 20 iterations of the modified 3D-3Way FRET algorithm showing improved axial resolution and devoid of false positive artifacts. (g) The MSE, DBSC and dDBSC were calculated at the xy plane shown for each iteration in order to measure convergence.

To validate the 3D-3Way FRET algorithm, reconstructions were performed on simulated data with defined fluorescence distributions. Similar to our previous work[8], we generated a spherical cell-like object with equal concentrations of fluorophores ([C], [Y], [R]) in the cytosol, and FRET complexes (E[CY], E[CR], E[YR]) in localized compartments (Fig 1A and S1 Fig, where, CFP = C, YFP = Y and RFP = R). Spectral mixing was applied by multiplying these distributions with the experimentally determined **B** resulting in the excitation and emission combinations (cc, cy, cr, yy, yr, rr; where the first letter denotes excitation and the second letter denotes emission) (Fig 1B). Convolution with a theoretical PSF produced a blurred noise-free estimate (Fig 1C). Photon noise was modeled using a Poisson distribution to produce the final simulated excitation/emission combinations; similar to those that would be captured on a widefield microscope (Fig 1D). Noise in this simulation was estimated to be SNR = 10 from the average intensity of the midplane (z = 45). The blurred and noisy images were unmixed with **B**^**-1**^ to calculate the N-Way FRET estimates without deconvolution; in this situation the SNR of the FRET signals was ~1.3 (Fig 1E), and the reconstruction was compared with this result to determine the performance of the algorithm.

**Fig 2:**
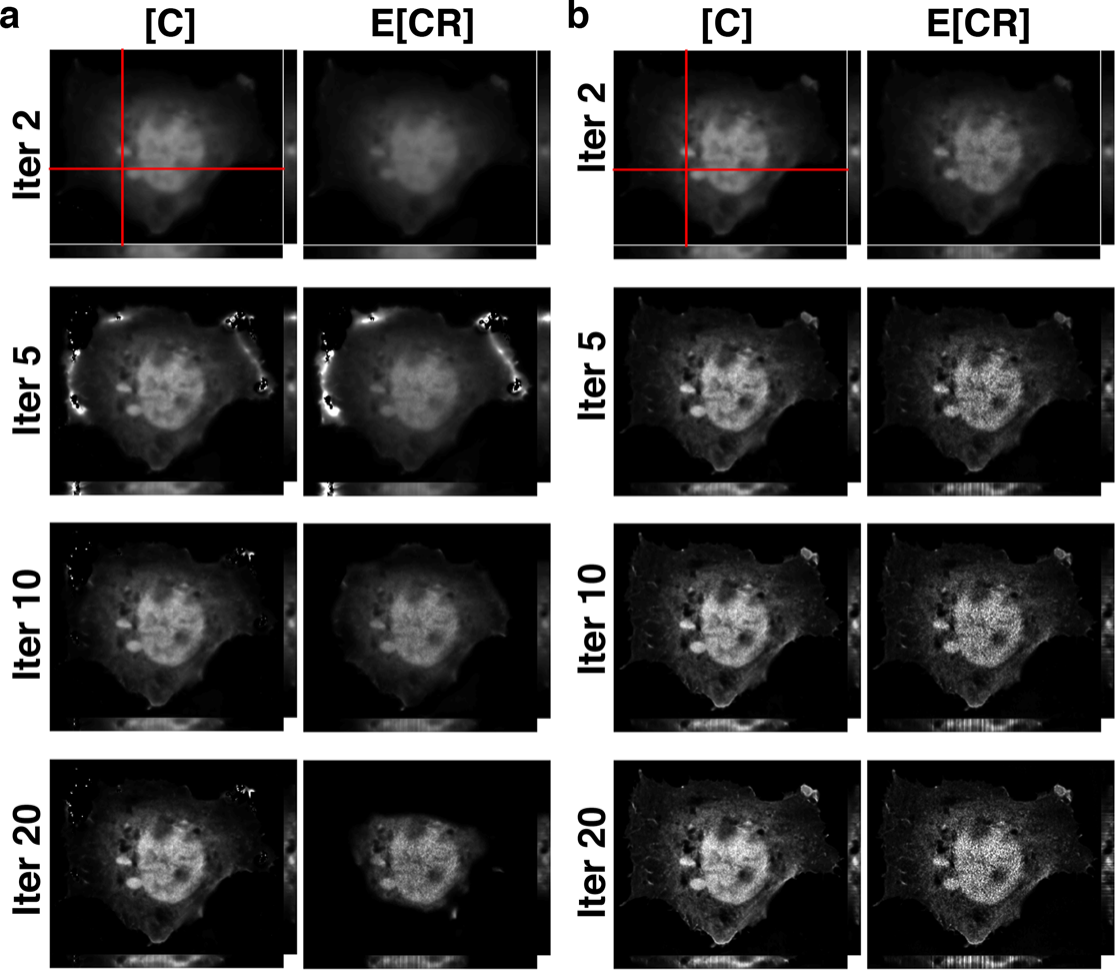
The modified algorithm (3D-3Way FRET) recovers total concentrations and apparent FRET efficiencies for linked fluorophores. (a) Cos7 cells expressing the triple linked construct RFP-CFP-YFP were imaged and reconstructed with 20 iterations of the original 3DFSR algorithm (only [C] and E[CR] are shown for clarity). Note that the E[CR] signal at the margin of the cell erodes away, until only the bright nucleus region remains. (b) The revised 3D-3Way FRET algorithm, properly reconstructs the images without the erosion that was evident in a.

We attempted to implement 3DFSR on the simulation, but the original 3DFSR algorithm failed to accurately reconstruct FRET signals for 3-way FRET. The edges of the object appeared to erode during the reconstruction (data not shown). These features were subtle because of the simple and symmetrical geometries of the simulated data. However, when 3DFSR was applied to actual cells expressing the linked polyprotein RFP-CFP-YFP, pronounced degradation of the image was observed (Fig 2 and S2A Fig). To overcome this limitation, we took advantage of the fact that N-Way FRET defined a spectral matrix **A** that is non-negative and could unmix FRET data into arbitrary fluorescence abundances of fluorophore and FRET signals (3). The fluorescence abundances (**x**) and concentrations and FRET efficiencies (**c**) are interchangeable per (4). Thus, we modified the algorithm such that the output of the line search (**c**^**k**^) was converted into fluorescence abundances (**x**^**k**^) (algorithm step 4), used for EM steps and then converted back into concentrations (**c**^**k+1**^) to complete the update (algorithm step 8). The resulting modified algorithm (3D-3Way FRET) properly reconstructed FRET signals for both simulated (Fig 1F) and experimental data (Fig 2B, S2B Fig).

Using the revised algorithm, the simulated data were reconstructed with 20 iterations of 3D-3Way FRET. The reconstructed estimates maintained correct values and spatial distributions; especially the FRET signals, which were nearly undetectable in the N-Way unmixed data (Fig 1E) were now restricted to the localized puncta with improved axial resolution (Fig 1F). The mean square error (MSE) between the estimate and the true value (initial model) was calculated at each iteration (Fig 1G) to measure convergence of the algorithm. Overall, the results for the total fluorophore concentrations asymptotically improve as seen by the reduction in the MSE. The MSE of the FRET signals improve as the algorithm progresses; however, they reach a minima around iteration 10 and then begin to diverge. Upon inspection of the reconstructed images, after 20 iterations the algorithm continues to fit finer and finer detail, leading to overfitting the data and corruption of the final estimates with noise. This divergence was seen previously with 3DFSR, and because it does not occur in the absence of noise suggests that convergence of the different channels is reached at different rates. Thus, to prevent overfitting a stopping criteria must be developed. Overall, the simulation results validated 3D reconstruction of 3-way FRET interactions and provides a powerful new method to improve the spatial resolution of 3D widefield FRET data.

The large size of the data and the computational requirements of 3D-3Way FRET drove us to optimize the computational algorithm in order to perform reconstructions at a faster rate. 3D-3Way FRET data are image tensors with 6 channels, and each z-stack was ~500x500x34. This corresponded to data stacks that were twice as large as those used in 3DFSR (3 image tensors). We analyzed the algorithm to find the rate-limiting step and attempted to optimize the algorithm speed. The slowest calculation per iteration was the convolution of the data with the PSF. Each iteration required 6–7 convolutions across all channels (depending on overrelaxation and refinement), resulting in about 42 total convolutions per iteration. The 3D-3Way FRET algorithm implemented an improved numerical procedure for the fast Fourier transform during the convolution calculations, which were parallelized onto 6 CPU cores. This change sped up the convolution calculation by ~100x. For our computer, this approach lead to the convolutions being faster than the overhead time required for parallelizing, distributing and gathering the data following convolution. With this improvement, all calculations could be performed on a single CPU core at the same speed as 6 CPU cores, suggesting that parallelization will only help if the data can be split across another dimension such as time for time-series movies. As a comparison, the average time for 20 iterations of 3D-3Way FRET was 20–30 minutes (6 image tensors), while 3DFSR required 5–12 hours to reconstruct data that is half the size (3 image tensors[8]). The major reduction in computational time makes increasing the number of iterations and exploring alternative stopping criteria tractable.

**Fig 3:**
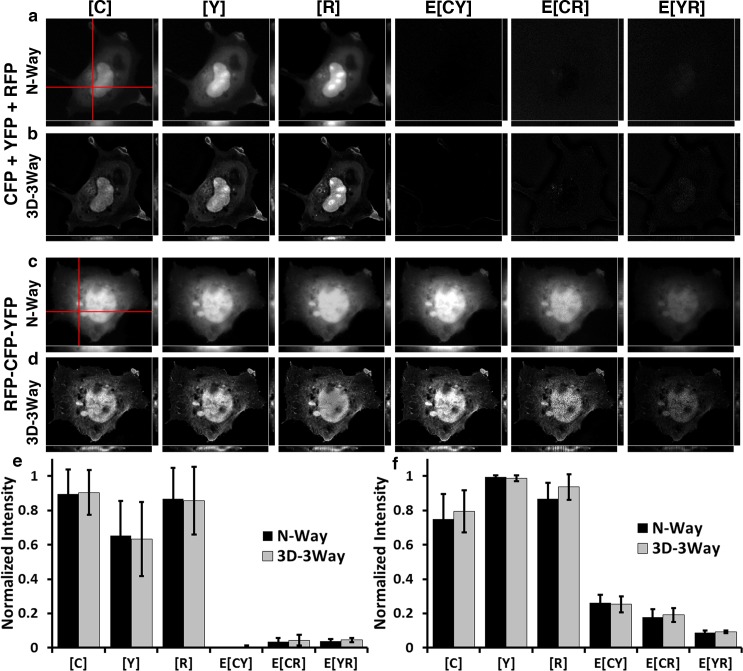
3D-3Way FRET improved the spatial distributions for free and linked fluorophores without introducing bias. Cos7 cells expressing cytosolic CFP, YFP, and RFP unmixed without deconvolution (a), and improved estimates following 20 iterations of 3D-3Way FRET (b). Cos7 cells expressing triple linked construct RFP-CFP-YFP unmixed without deconvolution (c), and following 20 iterations of 3D-3Way FRET (d). 3D data sets were collected for each cell with a resulting voxel sampling of 133nm X 133nm X 175nm. The images were directly unmixed by N-Way FRET (a and c) and the estimates following 20 iterations of the 3D-3Way FRET algorithm are shown in b and d. The red lines represent the plane taken for xz (shown below panels) and yz projections (shown to right of panels). The total fluorophore concentrations, [X], are co-scaled and E[X] are co-scaled to 30% of the total fluorophore concentrations. The reconstructed images (c and d) have improved resolution, especially the axial resolution as seen in the xz and xy projections. Quantification of fluorescence intensity over the entire 3D volume from CFP, YFP, and RFP (e) or RFP-CFP-YFP (f), unmixed with N-Way FRET (black bars) and also following 3D-3Way FRET (gray bars); error bars are standard deviation, N = 5 cells. 3D-3Way FRET intensities were compared to the corresponding N-Way FRET intensities, and there were no significant differences (p<0.05) using Two-way ANOVA followed by Tukey HSD post hoc comparison of means.

**Fig 4:**
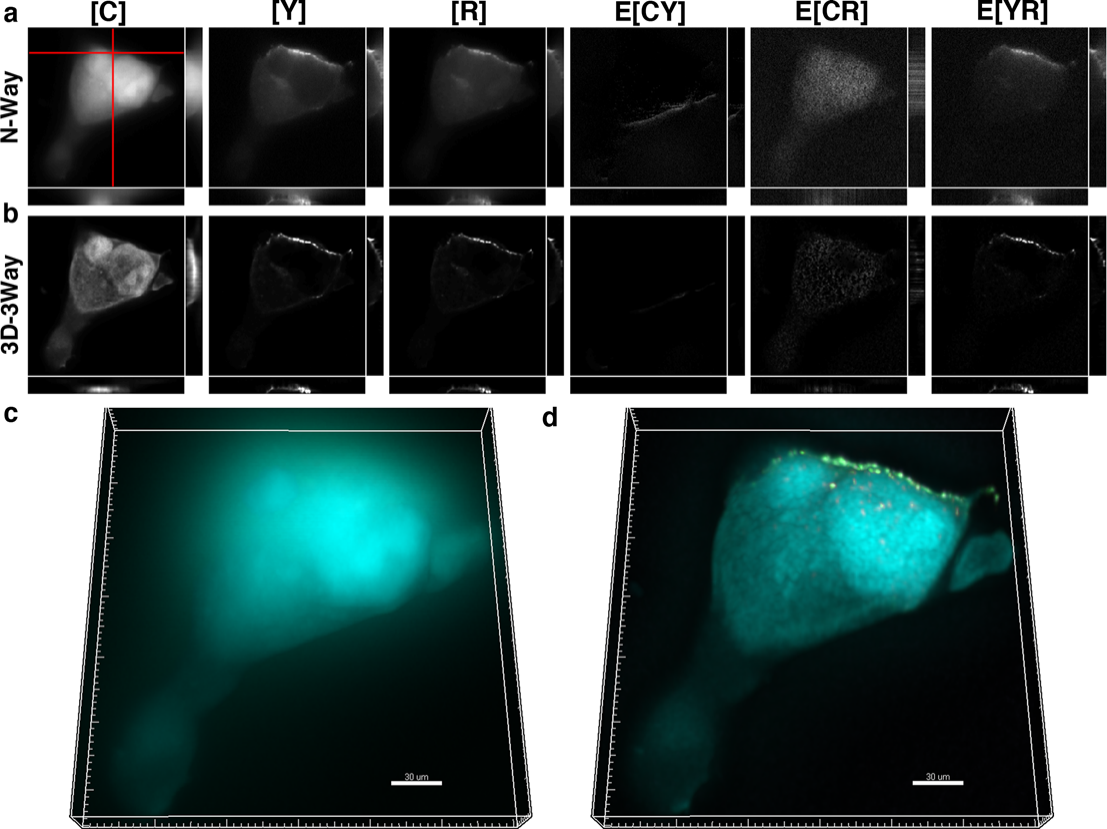
3D-3Way FRET provides improved estimates for the concentration and localization of HIV-Gag oligomerization in the presence of a noninteracting fluorophore. Cos7 cells expressing cytosolic CFP, YFP-Gag, and RFP-Gag were fixed and imaged 30 hrs post transfection. The oligomerization of Gag into punctate structures can be seen by direct application of N-Way FRET (a), and following 20 iterations of 3D-3Way FRET (b), the FRET complexes exist only in E[YR], are largely restricted to the puncta, and the axial resolution is greatly improved. 3D composite rendering of [C] (cyan) [Y] (green) and E[YR] (magenta) shown following N-Way unmixing (c), and following 3D-3Way reconstruction (d). The reconstructed image highlights the improved resolution.

**Fig 5:**
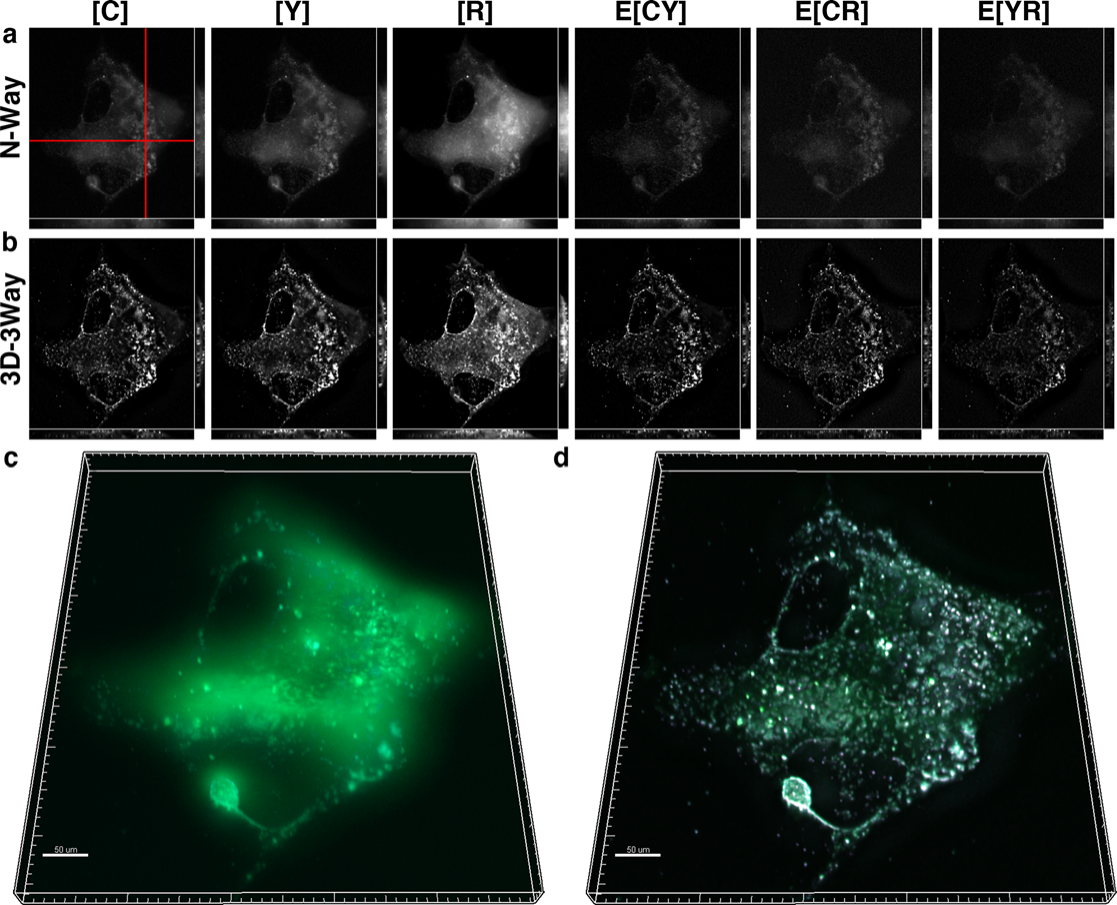
3D-3Way FRET provides improved estimates for the concentration and localization of HIV-Gag oligomerization in fixed cells. Cos7 cells expressing CFP-Gag, YFP-Gag, and RFP-Gag were fixed and imaged 30 hrs post transfection. The oligomerization of Gag into punctate structures can be seen by direct application of N-Way FRET (a). Following 20 iterations of 3D-3Way FRET (b) the FRET complexes are largely restricted to the puncta and the axial resolution is greatly improved. 3D composite rendering of [C] (cyan) [Y] (green) and E[CY] (magenta) shown following N-Way unmixing (c) and following 3D-3Way reconstruction (d). The reconstructed image highlights the improved axial resolution.

The MSE can only be used to measure convergence on simulated data because the true distribution is not known for real data, but required to measure the MSE. We explored stopping criteria that do not require knowing the solution a priori. We tested the differential based stopping criterion[13] (DBSC). In this statistic, the absolute difference between two consecutive estimates is quantified as a measure of convergence. The DBSC began to level off to different absolute values by the 10^th^ iteration, very close to the observed MSE minima for the FRET signals, but the DBSC traces were very noisy by comparison, (Fig 1G) making it difficult to define a single asymptotic stopping point. As a potential alternative, we measured the derivative of the DBSC (dDBSC) as a stopping criterion, here the rate of change approaches zero close to the minima of the MSE but, the large fluctuations in the dDBSC indicated that it may not be sufficiently robust to define a good stopping point. Thus, we present both DBSC and dDBSC in S3–S5 Figs for comparison and future algorithm development, but all reconstructions of experimental data were stopped based on visual inspection of the estimates and setting a fixed number of iterations (Figs 3–5).

We devised a series of test cases in which cells expressed FRET and non-FRET constructs to experimentally validate the 3D-3Way FRET reconstruction approach. Cells were imaged in 3D by capturing interlaced multiwavelength z-series. In all cases, N-Way FRET was used to unmix these images without deconvolution, thereby providing a reference point to which the reconstructed results could be compared. Following 3D-3Way reconstruction, the resulting images were compared to the N-Way FRET unmixed results to determine if spurious FRET signals or artifacts were observed and to gauge the axial resolution improvement by visual inspection. The following test cases were used to control the possible FRET interactions:

1. Three cytosolic non-interacting FPs (no FRET).
2. A linked polyprotein with equal stoichiometry of the 3 FPs and known FRET efficiency determined by acceptor photobleaching (intramolecular FRET between all FPs).
3. Non-interacting cytosolic CFP with YFP-Gag and RFP-Gag fusion proteins in which we expect FRET between YFP-Gag and RFP-Gag only [4,14].
4. Cell coexpressing CFP-Gag, YFP-Gag and RFP-Gag fusion proteins coexpressed (in which we expect intermolecular FRET between all FPs)[4].

These combinations define the minimum set of cases needed to test the algorithm for all 3-way FRET possibilities.

3D-3Way FRET was evaluated for its ability to recover FP distributions without producing artifacts that appear as FRET by imaging free FPs. COS7 cells expressing the cytosolic FPs CFP, YFP, and RFP, were imaged at a voxel size of 133nm x 133nm x 175nm and unmixed by N-Way FRET without deconvolution (Fig 3A). The FPs exhibited similar subcellular distributions, but do not interact with one another (E[CY], E[CR] and E[YR] are near zero, Fig 3E). The N-Way FRET unmixed results remained blurred as expected; this is especially evident in the xz and yz projections of Fig 3A. Following 20 iterations of 3D-3Way FRET, the in-focus plane features have sharper contrast and the axial resolution was enhanced (Fig 3B), while maintaining the same average intensities (Fig 3E). Thus, 3D-3Way FRET could recover non-FRET distributions without producing spurious artifacts.

3D reconstructions of samples containing FRET signals from single-chain biosensors were used to test the algorithms ability to recover FRET efficiency distributions. The RFP-CFP-YFP polyprotein ensures that the FPs will have the same cellular distributions, theoretically equal stoichiometry, and are held in close enough proximity for FRET to occur. The N-Way FRET unmixed data showed concentrations and apparent FRET efficiencies (Fig 3F) consistent with those measured by acceptor photobleaching on the same construct (E[CY] = 0.35+/- 0.00, E[CR] = 0.26+/- 0.01, E[YR] = 0.11+/-0.02, N = 4 cells). Additionally, the in-focus plane had several displacements in the cytosol by organelles that were subtle in the blurred images, but reconstructing the 3D data with 20 iterations of 3D-3Way FRET improved the resolution and highlighted these features (compare Fig 3C with 3D).

Importantly, the estimates recovered by 3D-3Way FRET have the same relative concentrations and FRET efficiencies as those estimated without 3D reconstruction by direct unmixing of the data by N-Way FRET (Fig 3E and 3F), which should only happen in when fluorophores and complexes have the same distributions throughout the cell. This highlights the accuracy of the algorithm and the ability to reconstruct FRET data without introducing a bias into the improved estimates. Thus, for a uniform distribution of fluorophores, 3D-3Way FRET accurately quantified FRET efficiencies of intramolecular interactions with improved 3D resolution.

The ability of 3D-3Way FRET to image intermolecular interactions in the presence of a non-interacting fluorophore was evaluated by coexpressing cytosolic CFP with YFP-Gag and RFP-Gag. We expect FRET only to occur between Gag fusion proteins near the plasma membrane at sites where viral-like particles form. The distribution of Gag molecules ([Y] and [R]) and FRET signals (E[YR]) were mainly found near the edge of the cell in the N-Way FRET unmixed result, but a small amount remained throughout the cytoplasm (Fig 4A). Additionally, the out-of-focus signal from cytosolic CFP obscured localized Gag puncta and FRET signals in the 3D rendered volume (Fig 4C). Following 20 iterations of 3D-3Way FRET, the fluorescence from FP-Gag fusions was restricted to the plasma membrane, and only there, was FRET measured between Y-Gag and R-Gag (E[YR]) (Fig 4B). Additionally, the sharpness of the cytosolic CFP distribution was improved following reconstruction (Fig 4D). The reconstruction shown in Fig 4 is representative of 5 reconstructions, which show similar improvements in spatial resolution and signal contrast. As a result, 3D-3Way FRET could be used to quantify localized pairwise protein interactions in the presence of a large pool of non-interacting molecules.

The algorithm was tested to quantify three intermolecular protein interactions. CFP-Gag was coexpressed along with YFP-Gag and RFP-Gag in order to quantify intermolecular interactions (E[CY], E[CR] and E[YR]) as viral-like particles formed. In this case, we expected similar fluorescence distributions at sites of viral assembly and FRET signals to localize to sites of viral assembly. The expected intermolecular interactions were quantified by unmixing with **B**^-1^ (Fig 5A). However, the spatial distributions of the interactions were poorly resolved with most of the FRET signal appearing as a diffuse pattern across the entire cell (Fig 5A and 5C). Following 20 iterations of 3D-3Way FRET, the FRET signals were highly restricted to punctate structures, and the images have high signal contrast (Fig 5B and 5D). The z projections of N-Way FRET unmixed data (Fig 5A) were very blurry and had limited information in comparison to the reconstructed estimates (Fig 5B) where individual puncta (the smallest of which are likely individual viral-like particles) became visible. The reconstruction shown in Fig 5 is representative of 5 reconstructions, which show similar improvements in spatial resolution and signal contrast. This confirms that 3D-3Way FRET reconstruction improved 3D distributions of protein interactions, and provides methods to quantify the spatial control of multi-protein complexes.

We explored possible stopping criteria that did not require knowledge of the true object by calculating the DBSC and dDBSC. In each test case the DBSC and dDBSC show a similar trend as in the simulation (Compare Fig 1G with S3–S5 Figs); both stopping criteria rapidly and asymptotically reduce after only a few iterations, well before convergence based on visual inspection. In particular, the dDBSC fluctuates around zero in fewer than 10 iterations in all cases, indicating very small differences between iterations. However, as the reconstruction approaches convergence by visual inspection there are no obvious changes in either statistic that signal a robust stopping point. We noticed that dDBSC highlights an oscillation of the estimates near convergence (S3 Fig) (as seen previously with 3DFSR). The oscillations were likely a result of the joint alternating LS and EM functionals that are minimized during each update in 3D-3Way FRET. This oscillation may be reduced by either using a fixed step size of 1 for each EM update, accurately calculating the EM step sizes from the likelihood functional, or using a penalized EM (computationally expensive[9,15]) rather than using overrelaxation from a numerical series, but this will require a greater number of iterations to reach convergence. However, the improved speed of reconstruction provides a good platform for implementation of more complex and robust stopping criteria.

## Conclusion

The 3D-3Way FRET approach presented here accurately reconstructs high-resolution 3D maps of protein-protein interactions in cells. The method was validated by simulation, linked constructs with known FRET efficiency and by imaging the oligomerization of HIV Gag proteins. In all of these tests, 3D-3Way FRET improved the 3D spatial distributions of free molecules and those in complexes, without introducing spurious FRET signals. Importantly, the reconstruction approach was robust for both intramolecular interactions with defined stoichiometry, and intermolecular interactions with unknown stoichiometry. However, there is room for further algorithm development. One persisting question for both 3DFSR and 3D-3Way FRET is that while the alternating LS and EM algorithm is highly robust, and performs very well under multiple conditions [8], we do not have a clear fundamental mathematical explanation for this. Second, in this work, we explored a possible stopping criterion; however, work is still needed to develop adequate convergence and stopping indicators.

Careful selection is needed when choosing fluorescent proteins for 3D-3Way FRET. Fluorophores that have good photostabilities and FRET efficiencies are necessary for imaging dim samples or time-series movies[12]. From our experience, it appears that the YFPs are the limiting fluorophores for this method owing to their limited photostability. Nonetheless, 3D-3Way FRET is a highly robust solution against noise, allowing for improved quality data to be reconstructed from lower signal data (therefore reducing sample photobleaching) thereby improving imaging of the spatiotemporal organization of cellular signaling pathways.

We have improved the computational speed of 3D-3Way FRET making the algorithm more accessible to a range of imaging situations and needs. Specifically, increasing the number of interactions simultaneously imaged or eliminating autofluorescence components requires larger datasets and greater computational requirements. However, the 2-log reduction in computational time greatly improves the usability of the algorithm, and provides a good platform for adding additional components or for testing appropriate stopping criteria. Additionally, 3D-3Way FRET uses the proper Poisson noise model providing a more statistically correct solution for FRET than the Gaussian noise model used in N-Way FRET and other spectral methods, albeit at a high computational requirement[4]. Thus, 3D-3Way FRET is capable of improving the spatial resolution of multiple protein-protein interactions on data collected by any microscopy approach capable of measuring FRET[8] (i.e. widefield, confocal, superresolution or multispectral imaging). These improvements make 3D-3Way FRET a valuable method for providing high-resolution maps of multiple protein-protein interactions throughout the 3D volume of cells.

## Supporting Information

S1 Fig Simulated fluorophore distributions and FRET interactions. (a) The 4.5 µm cell was simulated with a uniform fluorophore concentration in the cytosol, a void labeled as vacuole with no fluorophore, and 4 distinct spots that have mixtures of 2 and 3 FRET interactions. The mixtures of FRET signals in each spot are shown below. (b) The defined fluorophore distributions and localized FRET signals used for the simulation (Fig 1).
(TIF)

S2 Fig The modified 3DFSR algorithm (3D-3Way FRET) accurately recovers spatial distributions and FRET efficiencies for linked fluorophores. The data is associated with Fig 2, and is expanded to show the progression of all reconstructed images.
(TIF)

S3 Fig DBSC and dDBSC plots associated with data in Fig 3. The potential stopping criteria were calculated for an ROI of the xy plane shown in Fig 3.
(TIF)

S4 Fig DBSC and dDBSC plots associated with data in Fig 4. The potential stopping criteria were calculated for an ROI of the xy plane shown in Fig 4.
(TIF)

S5 Fig DBSC and dDBSC plots associated with data in Fig 5. The potential stopping criteria were calculated for an ROI of the xy plane shown in Fig 5.
(TIF)

S1 Movie 3D volume rendering of 3D-3Way FRET reconstruction. The movie shows the progression of the reconstruction of [C] (CFP-Gag) through iteration (time) for data associated with Fig 5.
(MPG)

## References

[1] Aoki K, Kamioka Y, Matsuda M. Fluorescence resonance energy transfer imaging of cell signaling from in vitro to in vivo: basis of biosensor construction, live imaging, and image processing. Dev Growth Differ. 2013 5;55(4):515–22. 10.1111/dgd.12039 23387795

[2] Jares-Erijman EA, Jovin TM. Imaging molecular interactions in living cells by FRET microscopy. Current Opinion in Chemical Biology. 2006 10;10(5):409–16. 16949332 10.1016/j.cbpa.2006.08.021

[3] Lakowicz JR. Principles of Fluorescence Spectroscopy. 3rd ed. New York: Springer Science+Business Media, LLC; 2006.

[4] Hoppe AD, Scott BL, Welliver TP, Straight SW, Swanson JA. N-Way FRET Microscopy of Multiple Protein-Protein Interactions in Live Cells. PLoS ONE. 2013;8(6):e64760 10.1371/journal.pone.0064760 23762252 PMC3675202

[5] Hoppe AD. Quantitative FRET Microscopy of Live Cells In: Shorte SL, Frischknecht F, editors. Imaging Cellular and Molecular Biological Functions. Springer; 2007 pp. 157–80.

[6] Toomre D, Bewersdorf J. A New Wave of Cellular Imaging. Annu Rev Cell Dev Biol. 2010 11 10;26(1):285–314. 20929313 10.1146/annurev-cellbio-100109-104048

[7] Shaw PJ. Comparison of Widefield/Deconvolution and Confocal Microscopy for Three-Dimensional Imaging In: Pawley J, editor. Handbook of Biological Confocal Microscopy. 3rd ed. New York: Springer Science+Business Media, LLC; 2006 pp. 453–67.

[8] Hoppe AD, Shorte SL, Swanson JA, Heintzmann R. Three-dimensional FRET reconstruction microscopy for analysis of dynamic molecular interactions in live cells. Biophysical Journal. 2008 7;95(1):400–18. 10.1529/biophysj.107.125385 18339754 PMC2426648

[9] Verveer PJ, Gemkow MJ, Jovin TM. A comparison of image restoration approaches applied to three-dimensional confocal and wide-field fluorescence microscopy. J Microsc. 1999 1;193(1):50–61. 12558687 10.1046/j.1365-2818.1999.00421.x

[10] Kaufman L. Maximum likelihood, least squares, and penalized least squares for PET. IEEE Trans Med Imaging. 1993;12(2):200–14. 18218408 10.1109/42.232249

[11] Low-Nam ST, Lidke KA, Cutler PJ, Roovers RC, van Bergen en Henegouwen PMP, Wilson BS, et al ErbB1 dimerization is promoted by domain co-confinement and stabilized by ligand binding. Nat Struct Mol Biol. 2011 11;18(11):1244–9. 10.1038/nsmb.2135 22020299 PMC3210321

[12] Scott BL, Hoppe AD. Optimizing fluorescent protein trios for 3-Way FRET imaging of protein interactions in living cells. Sci Rep. Nature Publishing Group; 2015 6 22;:1–13. 10.1038/srep10270 PMC4487001 26130463

[13] Szolgay D, Szirányi T. Optimal stopping condition for iterative image deconvolution by new orthogonality criterion. Electron Lett. 2011;47(7):442.

[14] Hogue IB, Hoppe A, Ono A. Quantitative fluorescence resonance energy transfer microscopy analysis of the human immunodeficiency virus type 1 Gag-Gag interaction: relative contributions of the CA and NC domains and membrane binding. J Virol. 2009 7;83(14):7322–36. 10.1128/JVI.02545-08 19403686 PMC2704781

[15] Kim J, An S, Ahn S, Kim B. Depth-variant deconvolution of 3D widefield fluorescence microscopy using the penalized maximum likelihood estimation method. Opt Express. 2013 11 18;21(23):27668–81. 10.1364/OE.21.027668 24514285

