# Supplementary figures and images for: Three-Dimensional Reconstruction of Three-Way FRET Microscopy Improves Imaging of Multiple Protein-Protein Interactions
Source: PLoS One. 2016 Mar 29;11(3):e0152401. doi: 10.1371/journal.pone.0152401 (PMC4811573; doi:10.1371/journal.pone.0152401)

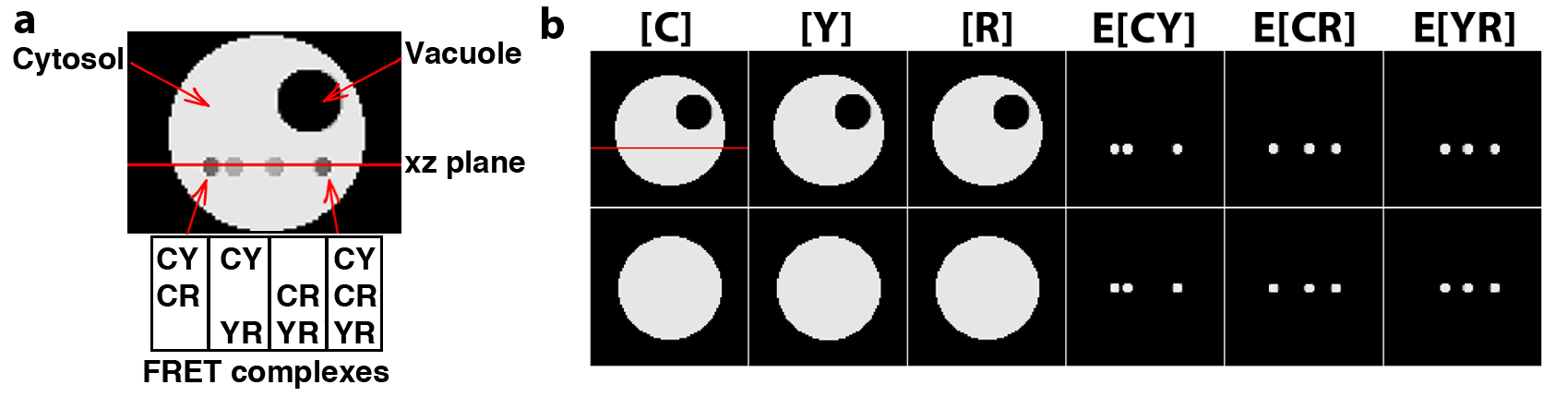

Supplement: S1 Fig — (a) The 4.5 µm cell was simulated with a uniform fluorophore concentration in the cytosol, a void labeled as vacuole with no fluorophore, and 4 distinct spots that have mixtures of 2 and 3 FRET interactions. The mixtures of FRET signals in each spot are shown below. (b) The defined fluorophore distributions and localized FRET signals used for the simulation (Fig 1). (TIF) [file pone.0152401.s001.tif]

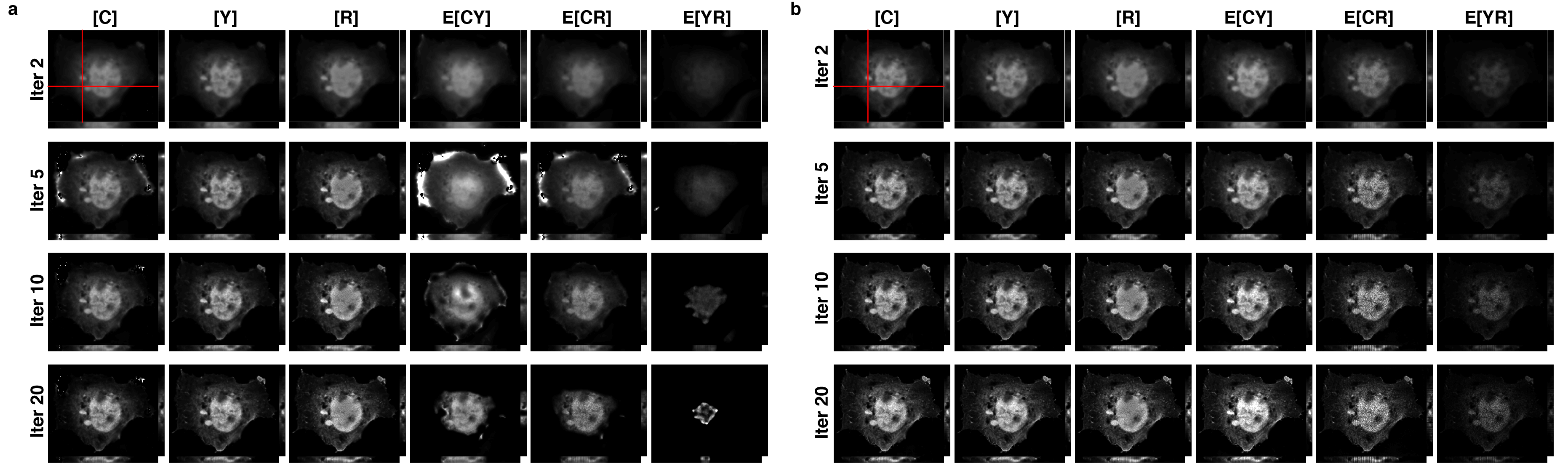

Supplement: S2 Fig — The data is associated with Fig 2, and is expanded to show the progression of all reconstructed images. (TIF) [file pone.0152401.s002.tif]

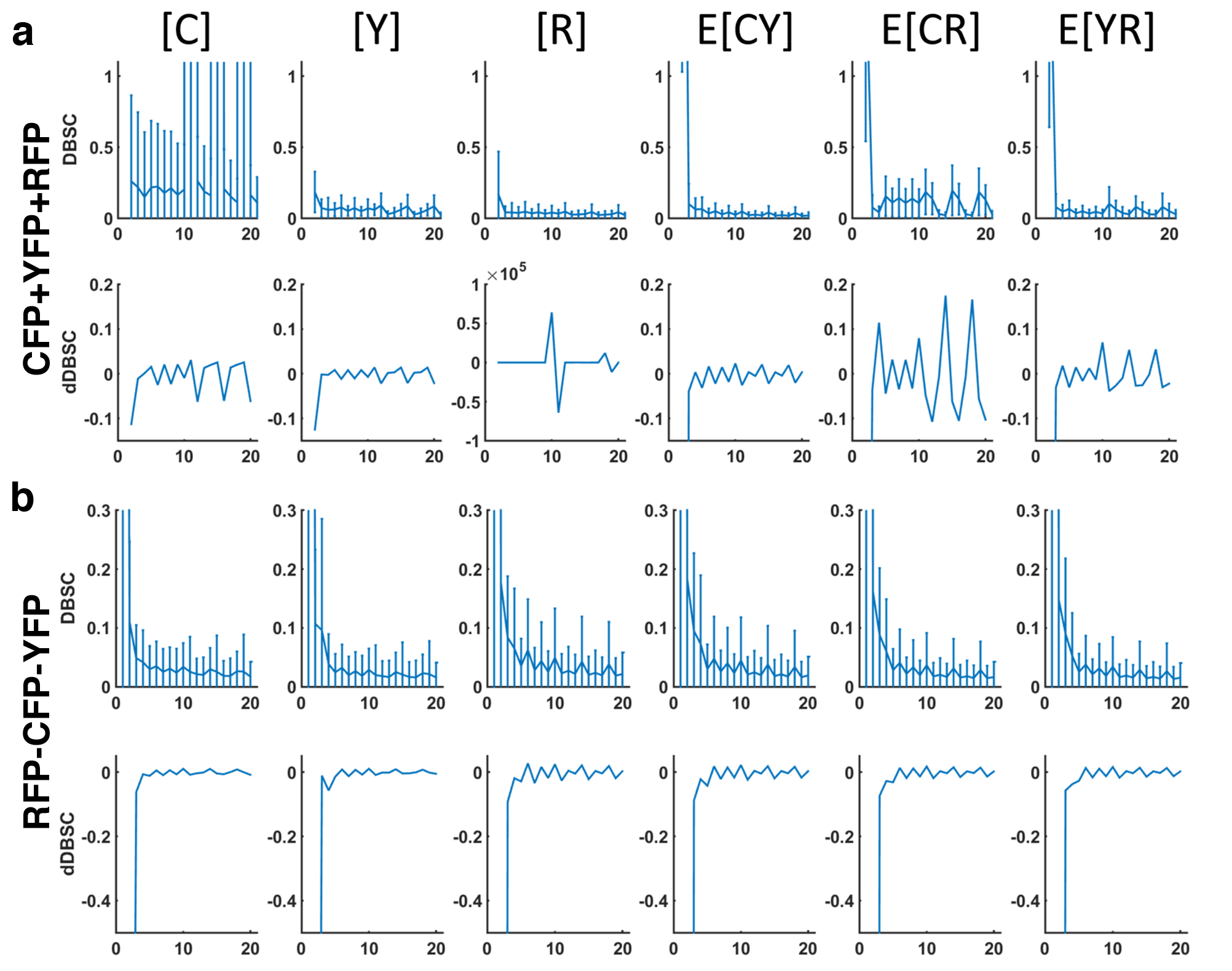

Supplement: S3 Fig — The potential stopping criteria were calculated for an ROI of the xy plane shown in Fig 3. (TIF) [file pone.0152401.s003.tif]

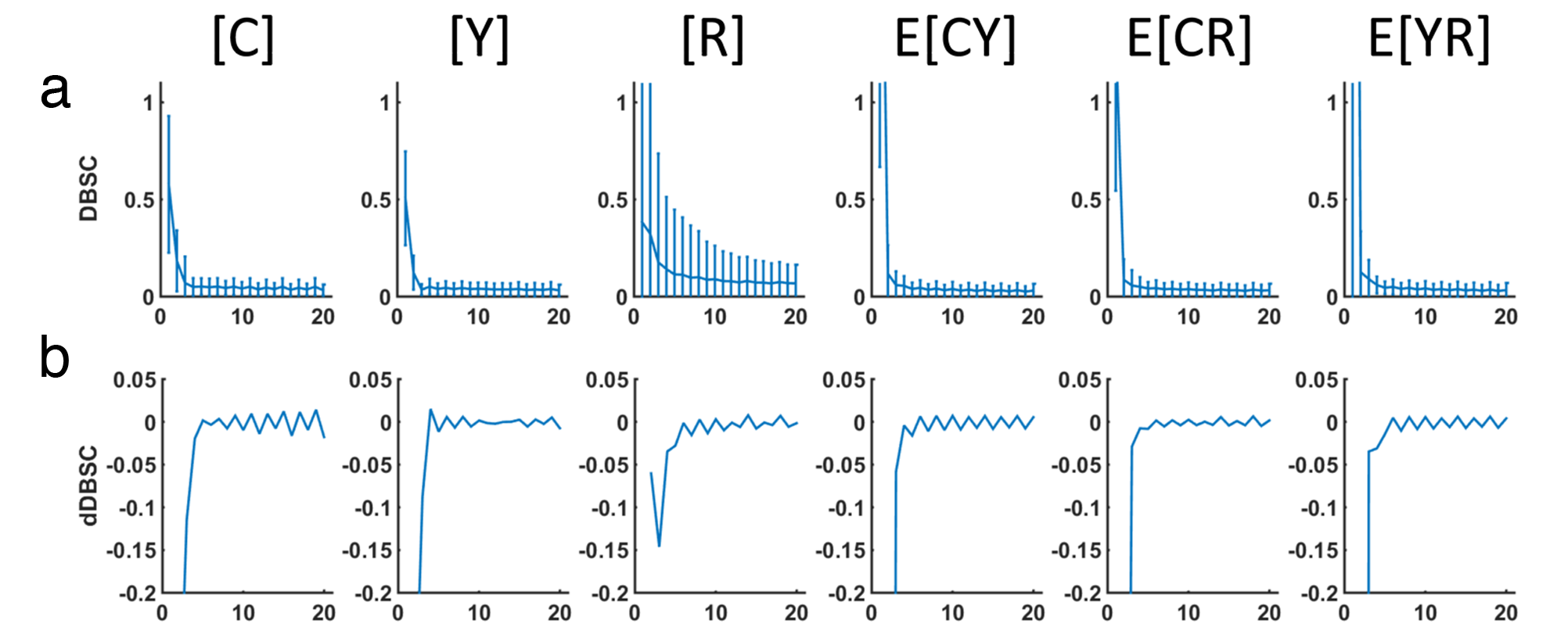

Supplement: S4 Fig — The potential stopping criteria were calculated for an ROI of the xy plane shown in Fig 4. (TIF) [file pone.0152401.s004.tif]

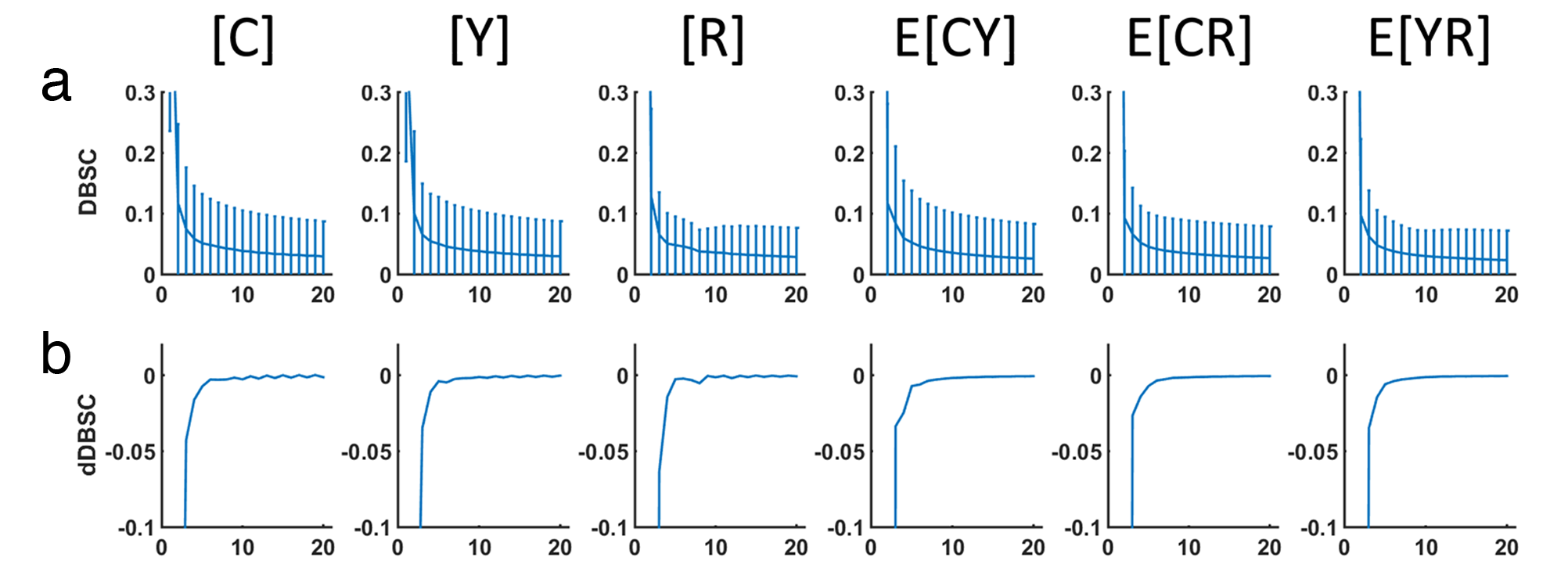

Supplement: S5 Fig — The potential stopping criteria were calculated for an ROI of the xy plane shown in Fig 5. (TIF) [file pone.0152401.s005.tif]
